# Anticholinergic burden in middle and older age is associated with lower cognitive function, but not with brain atrophy

**DOI:** 10.1111/bcp.15698

**Published:** 2023-03-09

**Authors:** Jure Mur, Riccardo E. Marioni, Tom C. Russ, Graciela Muniz‐Terrera, Simon R. Cox

**Affiliations:** ^1^ Lothian Birth Cohorts Group, Department of Psychology University of Edinburgh Edinburgh UK; ^2^ Centre for Genomic and Experimental Medicine, Institute of Genetics and Molecular Medicine University of Edinburgh Edinburgh UK; ^3^ Alzheimer Scotland Dementia Research Centre University of Edinburgh Edinburgh UK; ^4^ Edinburgh Dementia Prevention University of Edinburgh Edinburgh UK; ^5^ Division of Psychiatry, Centre for Clinical Brain Science University of Edinburgh Edinburgh UK; ^6^ Department of Social Medicine Ohio University Athens Ohio USA

**Keywords:** anticholinergic drugs, brain structural magnetic resonance imaging, cognitive ability, primary care

## Abstract

**Aims:**

The aim of this study is to estimate the association between anticholinergic burden, general cognitive ability and various measures of brain structural MRI in relatively healthy middle‐aged and older individuals.

**Methods:**

In the UK Biobank participants with linked health‐care records (*n* = 163,043, aged 40–71 at baseline), of whom about 17 000 had MRI data available, we calculated the total anticholinergic drug burden according to 15 different anticholinergic scales and due to different classes of drugs. We then used linear regression to explore the associations between anticholinergic burden and various measures of cognition and structural MRI, including general cognitive ability, 9 separate cognitive domains, brain atrophy, volumes of 68 cortical and 14 subcortical areas and fractional anisotropy and median diffusivity of 25 white‐matter tracts.

**Results:**

Anticholinergic burden was modestly associated with poorer cognition across most anticholinergic scales and cognitive tests (7/9 FDR‐adjusted significant associations, standardised betas (β) range: −0.039, −0.003). When using the anticholinergic scale exhibiting the strongest association with cognitive functions, anticholinergic burden due to only some classes of drugs exhibited negative associations with cognitive function, with β‐lactam antibiotics (β = −0.035, *P*_FDR_ < 0.001) and opioids (β = −0.026, *P*_FDR_ < 0.001) exhibiting the strongest effects. Anticholinergic burden was not associated with any measure of brain macrostructure or microstructure (*P*_FDR_ > 0.08).

**Conclusions:**

Anticholinergic burden is weakly associated with poorer cognition, but there is little evidence for associations with brain structure. Future studies might focus more broadly on polypharmacy or more narrowly on distinct drug classes, instead of using purported anticholinergic action to study the effects of drugs on cognitive ability.

**What is already known about this subject**

- Long‐term anticholinergic use is associated with a risk of dementia, but the evidence on the relationship with cognitive ability in healthy individuals is mixed. It is unclear if anticholinergic use is associated with measurable changes in brain structure before the onset of advanced age and dementia.
- The heterogeneity in previous studies may be due to differences in cognitive tests and anticholinergic scales used to measure the outcome and exposures, respectively, and in different effects of distinct classes of drugs.

**What this study adds**

- Our study suggests that while anticholinergic use according to most anticholinergic scales studied is associated with lower cognitive ability, the relationship holds only for some classes of drugs, especially β‐lactam antibiotics and opioids.
- In contrast to previous studies linking anticholinergic use to changes in brain structure in individuals with dementia, we found no such relationship in healthy individuals.

## 1 INTRODUCTION

Anticholinergic drugs (anticholinergics) are medicines thought to block muscarinic receptors. Their anticholinergic action is ascertained by consulting anticholinergic scales that assign potency scores to individual drugs; the combined score for an individual patient is the anticholinergic burden (AChB). Anticholinergics are commonly prescribed for a variety of conditions,^1^ and their transient side effects on cognition are well‐known.^2^, ^3^, ^4^, ^5^, ^6^ Moreover, their long‐term use in old age^7^ and middle age^8^, ^9^, ^10^, ^11^ has been associated with an increased risk of cognitive decline and dementia. It has been hypothesized^12^ that this relationship is due to central anticholinergic effects, affecting areas of the brain crucial for cognition.^13^, ^14^, ^15^ Therefore, a relationship might exist between AChB, cognitive ability and brain structure, even within the *normal* spectrum of cognitive functioning.

However, the existing evidence on this relationship is mixed. Most studies on anticholinergic prescribing in adults classify cognition as the absence *vs*. presence of a disorder or test separate cognitive modalities in isolation.^16^, ^17^ When measured this way, studies of AChB and cognitive ability often produce discordant results.^16^ There are reports of positive associations between anticholinergic use and executive function,^12^, ^18^, ^19^, ^20^, ^21^ associative learning,^22^ visual,^23^ episodic,^24^, ^25^ and short‐term memory,^26^ delayed and immediate recall,^27^ language abilities,^28^ visuospatial skills,^28^ attention,^28^ and reaction time [Correction added on 1 May 2023, after first online publication: The preceding sentence has been updated in this version.].^28^ However, some authors have found no evidence for delayed and immediate recall,^21^, ^22^, ^28^, ^29^ reaction time,^22^ executive function,^23^, ^27^ language abilities,^27^, ^29^ working memory,^25^, ^27^ processing speed,^25^ and implicit^28^ and semantic^25^ memory. Additionally, because anticholinergic scales sometimes include different drugs and score the same drugs differently,^30^ they could represent another source of variation in reported findings.

It has been suggested that global composites of cognitive functioning might be more sensitive to subtle cognitive changes.^16^ Individual test scores contain more random noise, and the results can limit generalisability and contribute to inconsistency among studies. By contrast, general cognitive ability (sometimes referred to as general intelligence or *g*) represents shared variation across cognitive domains, is predictive of various social outcomes,^31^ health outcomes,^32^, ^33^ mortality,^34^ and is referenced in widely utilized diagnostic manuals.^31^ Analysing large samples on multiple anticholinergic scales can further strengthen the reliability of the results.

Past studies have demonstrated associations between cognitive ability and several measures of brain structural magnetic resonance imaging (MRI). While the effect sizes have varied depending on the sample characteristics and cognitive tests used, they have usually ranged from *r* = 0.2 to  0.3.^35^ One review found evidence for cross‐sectional and longitudinal associations between global cognition and total brain size, global grey matter and hippocampal volume.^36^ An analysis conducted on the UK Biobank sample found correlations between general cognitive ability and total brain volume, functional anisotropy (FA), mean diffusivity (MD) and several regional cortical volumes, especially those in the frontal lobe. Additionally, the authors found associations for multiple subcortical structures, especially the thalamus.^37^ However, little is known about the neural correlates of potential anticholinergic‐related cognitive decline.

To our knowledge, 4 studies^8^, ^12^, ^38^, ^39^ to date have assessed the relationship between these brain measures and regular anticholinergic use. While each study reported on associations between anticholinergic use and various metrics of brain structure and function, replication studies in larger samples are required. Furthermore, research is needed to probe potential differences between anticholinergic scales and between different classes of anticholinergics when exploring associations with cognitive function and cerebral correlates.

In our study—conducted using the UK Biobank—we calculated a latent factor of general cognitive ability (*g*) and utilized MRI‐imaging measures and prescriptions linked from primary care, to study the association between AChB, *g* and various brain structural MRI measures. Our goals were to assess: (i) whether there existed differences between anticholinergic scales and (ii) between drug classes in the association of AChB and cognitive ability; and (I ii) whether potential associations between AChB and cognitive ability were reflected in brain MRI measures, including brain atrophy, the volume of various cortical and subcortical brain structures, and measures of white matter microstructure. Based on previous findings, we hypothesised AChB to negatively associate with *g*, total brain volume, and the volumes of prefrontal cortical areas, the thalamus and hippocampus.

## 2 METHODS

### 2.1 Sample

UK Biobank^40^ is a prospective study whose participants were recruited between 2006 and 2010 when they were aged 37–73 years. During the initial assessment, demographic and lifestyle questionnaires, physiological measurements and cognitive tests were administered. A subset of participants later underwent MRI structural imaging and additional cognitive testing. For ~230 000 participants, data on issued prescriptions and diagnoses are available. The diagnoses used were sourced from self‐reported data, primary care and secondary hospital care. Self‐reported data were provided at the time of the assessments, while data from primary care and secondary hospital care are available until August 2017 and March 2021, respectively. Prescriptions are complete until May 2016 and were sourced from primary care. The prescription entries contained names and dates of drugs prescribed by general practitioners and the (mostly region‐specific) suppliers of the prescription data. For the variables described below, we provide specific Field IDs (and links to the descriptions page for each field) in Table S1.

### 2.2 Cognitive ability

During the baseline assessment, most participants completed tests measuring visual declarative memory (Pairs Matching), processing speed (Reaction Time), with a subsample also completing tests of working memory (Numeric Memory), prospective memory (Prospective Memory), and verbal and numerical reasoning (Fluid Intelligence). During the imaging assessment, another subset of participants completed the above tests again, in addition to tests of executive function (Trail Making A and B, Tower Rearranging), verbal declarative memory (Paired Associate Learning), nonverbal reasoning (Matrix Pattern completion), crystallized ability (Picture Vocabulary) and another on processing speed (Symbol Digit Substitution; Table S2). Analyses of their psychometric properties in this sample have been reported previously.^41^, ^42^ We fitted a confirmatory factor analysis in a structural equation modelling (SEM) framework to calculate *g* from the cognitive tests (Figure S1 and Table S3), yielding 2 separate values, 1 for each assessment visit. SEM has been used to calculate *g* in UK Biobank before^37^, ^43^; the proportional variance explained in our study is smaller (23% for the baseline assessment, 28% for the imaging assessment) than in prior work in UK Biobank that used fewer cognitive tests.^37^ For participants for whom this was possible, *g* from the imaging assessment was used in our analyses.

### 2.3 Brain imaging

Since 2014, UK Biobank has been enhancing the dataset with imaging data that includes brain MRI.^40^, ^44^ It consists of imaging‐derived phenotypes, whose acquisition and quality control have been previously described.^45^ Briefly, brain imaging data were obtained at 4 data collection sites (Cheadle, Newcastle, Reading and Bristol; all UK) using 3 identical scanners (3T Siemens Skyra), with a standard Siemens 32‐channel receive head coil. Preprocessing and quality control were undertaken by the UK Biobank research team according to published procedures.^45^ Our analyses included total brain volume, brain volumes of 68 cortical areas, 14 subcortical structures, FA and MD of 25 white matter tracts. The measures of brain volume were corrected for head size by multiplication with the T1‐based scaling factor (UK Biobank field ID 25000). The brain regions and white matter tracts used in the study are depicted in Figure S2.

### 2.4 Anticholinergic burden and drug classification

Anticholinergic scales typically score drugs on a 0–3 ordinal scale, with a higher score indicating greater anticholinergic potency. We considered 15 anticholinergic scales—13^28^, ^46^, ^47^, ^48^, ^49^, ^50^, ^51^, ^52^, ^53^, ^54^, ^55^, ^56^, ^57^ were based on our previous analyses^1^ while 2 scales^58^, ^59^ were identified through a recent review^7^ (Table S4). Three scales^47^, ^50^, ^56^ were modified to include newer drugs.^1^, ^60^ One scale^52^ was modified so that drugs with *improbable anticholinergic action* were assigned an anticholinergic burden of 0.5 as was done before.^1^ Using the British National Formulary (https://bnf.nice.org.uk/, last accessed on 11 March 2021), we replaced brand names with generic names. Combination prescriptions containing several anticholinergics were each separated to yield multiple prescriptions, each containing a single anticholinergic. Each prescription was then assigned a potency score from each anticholinergic scale. For analysis, the cumulative AChB was calculated by summing the AChB scores across all prescribed drugs in the sampling period. The sampling period excluded the year preceding the UK Biobank assessment to avoid acute effects of drugs. Prescriptions of drugs with ophthalmic, otic, nasal or topical routes of administration were all assigned an anticholinergic score of 0, as previously reported.^1^, ^54^, ^55^, ^56^, ^57^ Each drug was assigned to a class in the WHO Anatomical Therapeutic Chemical (ATC) Classification system (https://www.whocc.no, last accessed on 11th March 2022)^61^ that categorizes drugs in a 5‐level hierarchy. In our analyses, the first (anatomical main group) and third (pharmacological subgroup) levels were used.

### 2.5 Data preparation

Prescriptions issued before 2000 and after 2015 were removed due to low ascertainment and incomplete annual data, respectively.^1^ Participants with a diagnosis of diseases that may affect brain structure or cognitive ability were removed. The data‐cleaning process is depicted in Figure S3. Outliers for numerical variables were defined as values lying 4 or more standard deviations or interquartile ranges beyond the mean or median, whichever was most appropriate according to the distribution. The total number of prescribed drugs and the AChB scores were strongly right‐skewed due to the high numbers of zero values. For these variables, zeroes were removed before identifying outliers. All outliers were removed before analysis. Model assumptions were mostly met, but some models exhibited non‐normality in the distribution of residuals (Figure S4).

### 2.6 Modelling

We applied principal component analysis to tract‐specific FA and MD and used the first principal component to compute the *general* FA and MD (*g*FA and *g*MD), accounting for 44 and 50% of the variance, respectively. The standardized loadings and proportional variance for *g*FA and *g*FA are presented in Figure S5 and Table S5. We used linear regression models to estimate the association between AChB, cognitive ability and brain structure. To compare anticholinergic scales, we first modelled the association between *g* and AChB for each scale separately. This was later repeated for total brain volume as the outcome. The scale exhibiting the strongest association with *g* was selected for subsequent analyses. Second, we modelled the effects of AChB due to different drug classes on *g* and total brain volume. Finally, we computed the associations between AChB and the results from 9 cognitive tests, the volumes of 68 cortical areas, 14 subcortical areas, *g*FA and *g*MD, and FA and MD of 25 white matter tracts. We also conducted 2 sensitivity analyses. First, we repeated the analyses on the association between AChB and *g* including only the year preceding the UK Biobank assessment to calculate AChB. Second, we computed the association between AChB according to each scale and *g*, while including an interaction term between AChB and age at assessment.

Each model was corrected for potential confounders, which included age at assessment, number of years over which the cumulative AChB was calculated, number of prescribed nonanticholinergics (different for each anticholinergic scale), data supplier of prescriptions (region‐specific—2 for England, and 1 each for Scotland and Wales), socioeconomic deprivation (higher values correspond to greater deprivation; range: −6.3–11.0),^62^ smoking status (nonsmoker, previous smoker, current smoker), frequency of alcohol consumption (daily or almost daily; 3 or 4 times a week; once or twice a week; once to 3 times a month; only on special occasions; never), level of physical activity (strenuous; moderate; mild),^63^ body mass index (kg/m^2^), *APOE*‐carrier status, comorbidities count before the first assessment date (total number of distinct diagnoses codes), history of mood disorders, anxiety disorders, schizophrenia, diabetes, hypercholesterolemia, hypertension and myocardial infarction before the assessment date. *APOE*‐carrier status was defined through the *APOE* genotype, which is based on the nucleotides at SNP positions rs239358 and rs7412. Participants were denoted as ε2, ε3, or ε4 carriers, if they carried the ε2/ε2 or ε2/ε3 haplotype, ε3/ε3 or ε1/ε3 or ε2/ε4 haplotype, or ε3/ε4 or ε4/ε4 haplotype, respectively. Smoking status, alcohol consumption, physical activity, body mass index and genotype were ascertained at each of the 2 UK Biobank assessments; socioeconomic deprivation was ascertained during the baseline assessment.

When comparing anticholinergic scales, 2 additional models were run for which polypharmacy was the main predictor. The first of these models (*Polypharmacy* model) controlled for the same covariates as above, and the second (*Polypharmacy plus* model) further controlled for the total number of anticholinergics according to any scale. The models where a measure of brain imaging was the main outcome, were in addition to the covariates above controlled for age^2^, age*sex, age^2^*age, head position in the MRI‐scanner (3 coordinates), ethnicity and assessment centre. The template for the linear models is described in Text S1. Results are presented for models before the adjustment for polypharmacy and after adjustment for polypharmacy. Unless explicitly stated otherwise, the results refer to the fully adjusted models.

In analyses where a single anticholinergic scale was used (as opposed to comparing several scales), AChB was calculated using the scale by Durán *et al*.,^52^ as it exhibited the strongest association with *g* (see Section 3). All numerical variables were normalized to have a mean of 0 and a standard deviation of 1. When several independent models were run to predict the same outcome, *P*‐values were corrected for multiple comparisons using the false discovery rate (FDR).^64^ Otherwise, the *P‐*threshold of 0.05 was used. Results are reported as standardized betas (β) and plotted with confidence intervals (CIs) adjusted for multiple comparisons (based on the Z‐values of the quantile for the standard normal distribution for the FDR‐adjusted *P*‐values). All data cleaning and modelling were performed using R version 4.2.1 and Python version 3.9.7.

## 3 RESULTS

### 3.1 Sample

After removing outliers, among the 163 043 participants in our sample, ~140 000 and ~14 000 data points (exact value depended on the model) were available for analyses of cognitive ability and brain imaging, respectively. The demographic‐ and lifestyle variables are presented in Table 1. While the imaging sample was older, the distribution of other variables was similar to the rest of the sample (Table S6). In the period from 2000 to the year before the initial assessment, anticholinergics—depending on the anticholinergic scale—represented between 4.3 and 24.1% of prescriptions, with between 11.3 and 40.7% of participants prescribed an anticholinergic at least once (Table S7). We have previously characterized anticholinergic prescribing and its longitudinal trends in UK Biobank in detail.^1^

**TABLE 1:** Demographic and lifestyle characteristics of the sample at the baseline assessment after the removal of outliers.

Variable	Level	Median (IQR) or *n* (%)	N missing
Age (years)		58.3 (12.8)	
Sex	Male	72 184 (44.3)	
	Female	90 859 (55.7)	
Deprivation (z‐score)		−2.3 (3.9)	174
Alcohol consumption	Daily or almost daily	32 244 (19.8)	326
	Three or 4 times a week	38 472 (23.6)	
	Once or twice a week	43 328 (26.6)	
	Once to 3 times a month	18 402 (11.3)	
	Only special occasions	18 045 (11.1)	
	Never	12 316 (7.6)	
Smoking	Current smoker	16 048 (9.9)	772
	Previous smoker	55 642 (34.3)	
	Non‐smoker	90 581 (55.8)	
Physical activity	Strenuous	16 531 (10.8)	10 707
	Moderate	97 776 (64.2)	
	Light	38 029 (25.0)	
BMI (kg/m^2^)		26.8 (5.8)	1029
Data provider	England (Vision)	14 393 (8.8)	
	Scotland	9571 (5.9)	
	England (TPP)	122 120 (74.9)	
	Wales	16 959 (10.4)	
Mood disorder		23.926 (14.7)	
Anxiety disorder		15.572 (9.6)	
Schizophrenia		590 (0.4)	
Myocardial infarction		7335 (4.5)	
Diabetes		14 477 (8.9)	
Hypercholesterolemia		29 994 (18.4)	
Hypertension		54 124 (33.2)	
Number of prior comorbidities		86 (98)	65
Polypharmacy		34 (96)	
*APOE* carrier	ε2	20 549 (12.9)	3871
	ε3	98 084 (61.6)	
	ε4	40 539 (25.5)	

*Note*: Polypharmacy is the total number of prescriptions issued over the sampling period (differs among participants; range: 1–16 years). Sex, deprivation, alcohol consumption, smoking, physical activity and BMI are self‐reported or based on measurements during the baseline assessment. The variables are not scaled.

Abbreviations: BMI: body mass index; IQR: interquartile range; TPP: The Phoenix Partnership.

### 3.2 AChB and cognition

**FIGURE 1:**
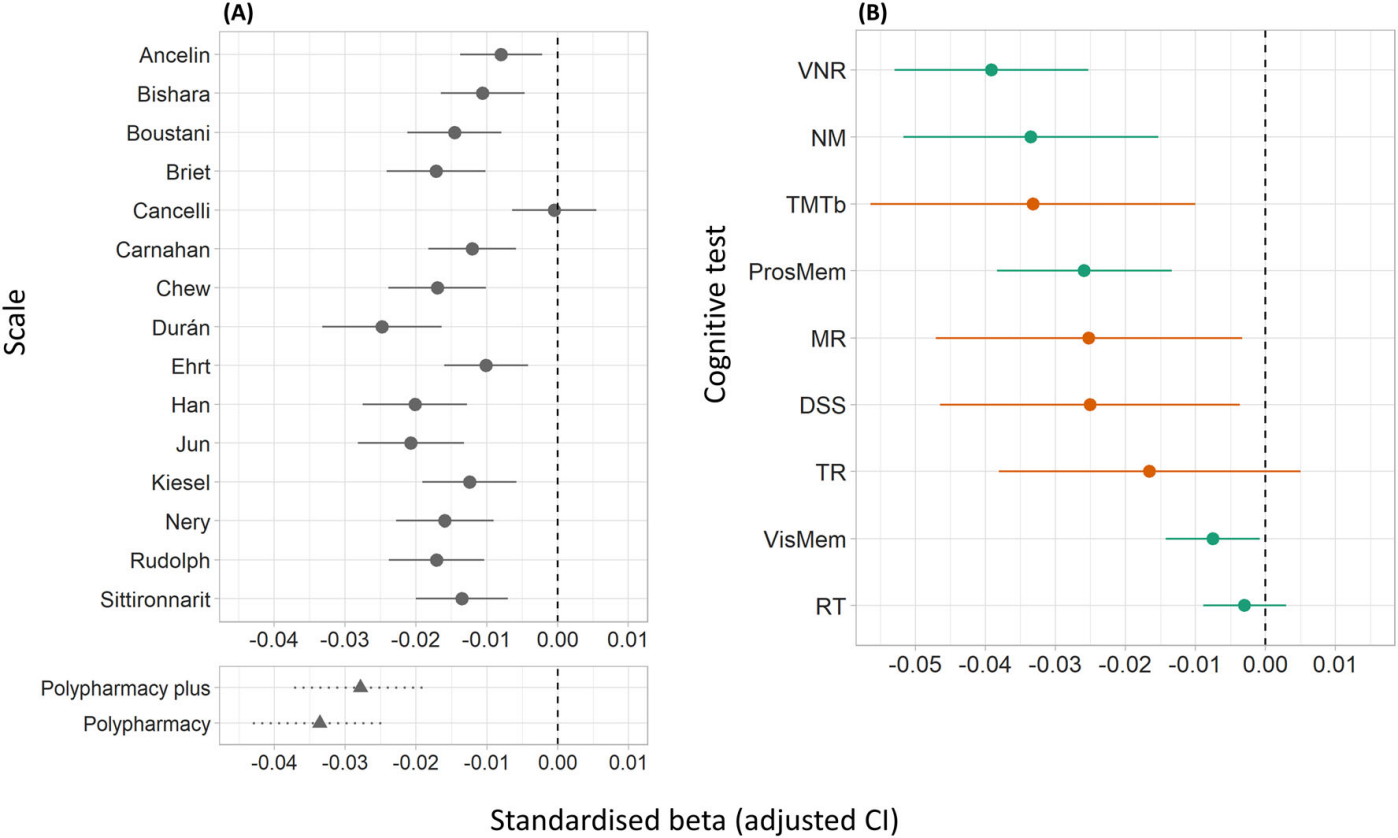
Associations between anticholinergic burden (AChB) and general cognitive ability for each anticholinergic scale (A) and associations between AChB according to the scale by Durán *et al*.^52^ and each cognitive test included in the calculation of general cognitive ability (B). Results are displayed as standardized βs. (A) The y‐axis indicates the main predictor for each model; in the upper panel, this was the AChB according to different anticholinergic scales; in the bottom panel, this was drug count (i.e., polypharmacy, controlled for in 2 different ways; see main text for details). (B) The y‐axis indicates the cognitive test used as the outcome. The colours refer to when the test was taken, with green indicating assessment at baseline and orange indicating assessment during the imaging visit.

When polypharmacy was not included as a control variable, all the tested anticholinergic scales exhibited significant negative associations with *g* (Table S8). The scales by Durán *et al*.^52^ and by Cancelli *et al*.^49^ showed the strongest (β = −0.032, *P*_FDR_ < .001) and weakest (β = −0.009, *P*_FDR_ < .001) effects, respectively. When the models were additionally corrected for polypharmacy, the median effect size of AChB across scales was reduced by 31%, but associations of all anticholinergic scales except the scale by Cancelli *et al*.^49^ (β = −4.4 × 10^−5^, *P*_FDR_ = .88) remained significant (Figure 1A, Table S8). The scale by Durán *et al*.^52^ retained the strongest association (β = −0.025, *P*_FDR_ < .001; Table S9). When the predictors were not standardized, this effect size corresponds to an at most 0.0017 decrease in *g* when AChB is increased by 1 standard deviation. The main predictors of each polypharmacy model also exhibited negative correlations with cognition, (*Polypharmacy*: β = −0.034 *P*_FDR_ < .001; *Polypharmacy plus*: β = −0.028, *P*_FDR_ < .001). The number of anticholinergics included in a scale was positively correlated with the strength of the observed effect when uncorrected for polypharmacy (*R* = .70, *P* = .004) and when corrected for polypharmacy (*R* = .60, *P* = .02, Figure S6). I.e., the more drugs were identified as anticholinergic by an anticholinergic scale, the better predictor the scale was of lower *g*.

When a separate model was run for each cognitive test, AChB exhibited negative associations for each test. Among the cognitive tests, 7/9 were significant; Fluid Intelligence showed the strongest effect (β = −0.039, *P*_FDR_ < .01) and Reaction Time (β = −0.0030, *P*_FDR_ = .33) exhibited the weakest effect (Figure 1B, Table S10).

**FIGURE 2:**
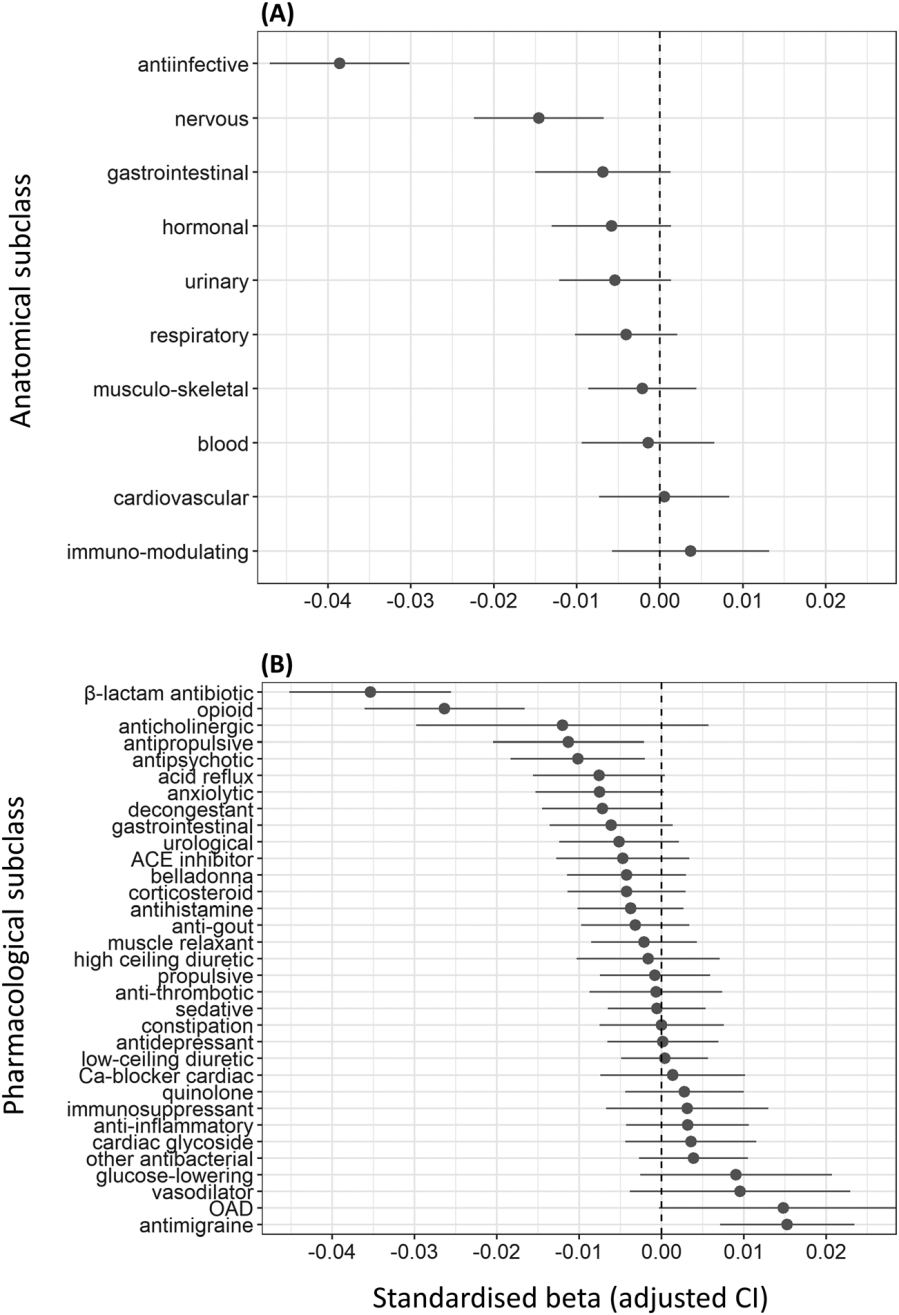
Associations between anticholinergic burden according to the scale by Durán *et al*.^52^ due to different classes of drugs on the 1 hand and general cognitive ability on the other. Results are displayed as standardized βs. (A) Classification of drugs based on anatomical class; (B) Classification of drugs based on pharmacological subclass. Classes containing drugs that were together prescribed to too few participants (<100) were not included in the models.

When testing for the effects of drug classes, we found only limited instances in which higher AChB was associated with lower *g* (Figure 2, Table S11). Among the pharmacological classes, AChB due to drugs for migraine (β = 0.015, *P*_FDR_ < .001) showed positive associations with *g*. AChB due to most other drugs exhibited negative associations with *g*, with β‐lactam antibiotics (β = −0.035, *P*_FDR_ < .001) and opioids (β = −0.026, *P*_FDR_ < .001) showing the strongest effects, corresponding to respectively 0.033 and 0.010 decreases in the unstandardized *g* for each increase of AChB by 1 standard deviation.

### 3.3 ACB and brain‐imaging measures

**FIGURE 3:**
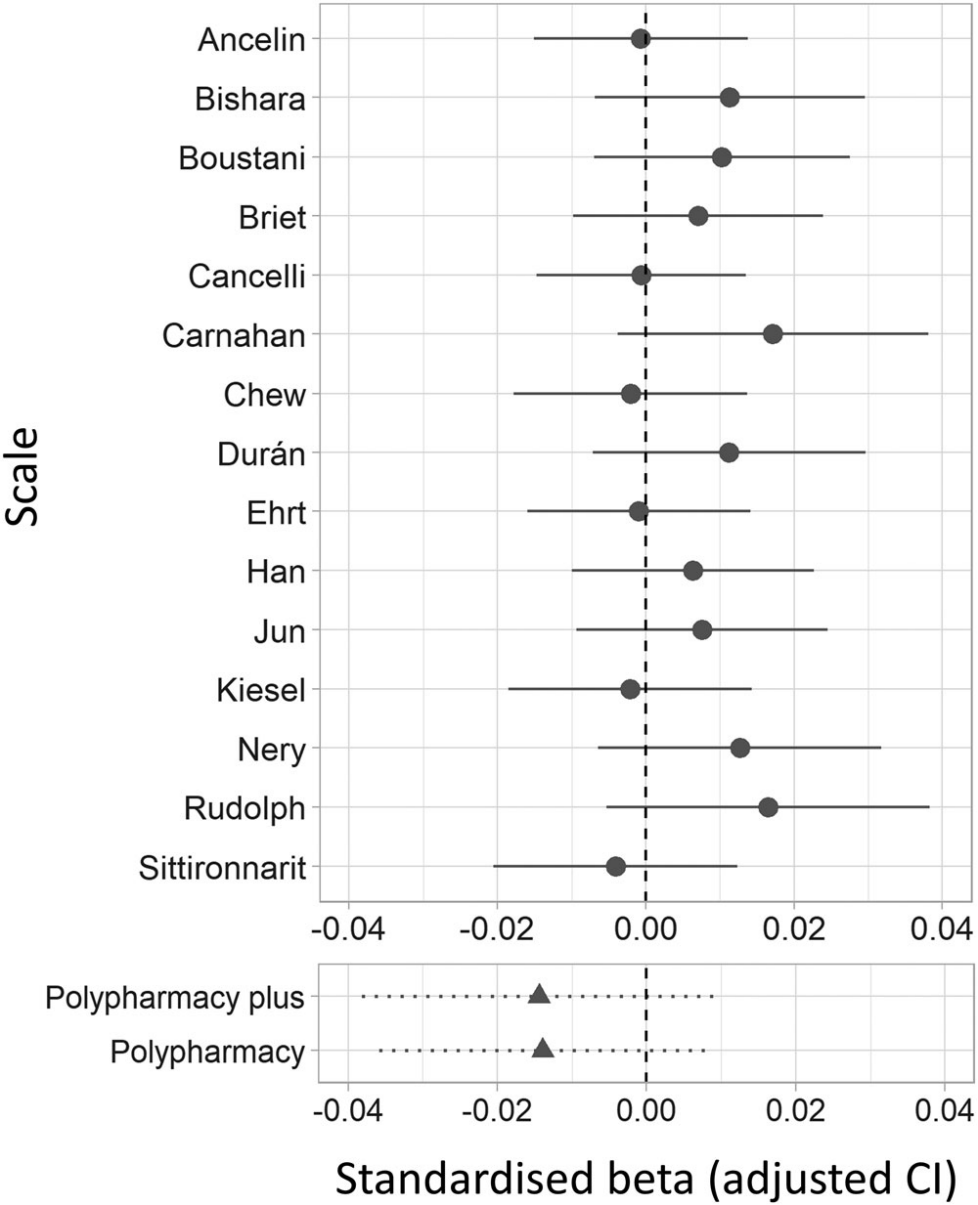
Associations between anticholinergic burden and brain atrophy for each anticholinergic scale. The y‐axis indicates the main predictor for each model; in the upper panel, this was the anticholinergic burden according to different anticholinergic scales; in the bottom panel, this was drug count (i.e., polypharmacy, adjusted for covariates in different ways; see main text for details). Results are displayed as standardized βs.

AChB was not associated with brain atrophy irrespective of the anticholinergic scale used (range of β = −0.004–0.017, *P*_FDR_ ≥ .21). While there were minor differences between the predictive power of different scales, the CIs overlapped across scale models and polypharmacy models (Figure 3, Tables S12 and S13).

**FIGURE 4:**
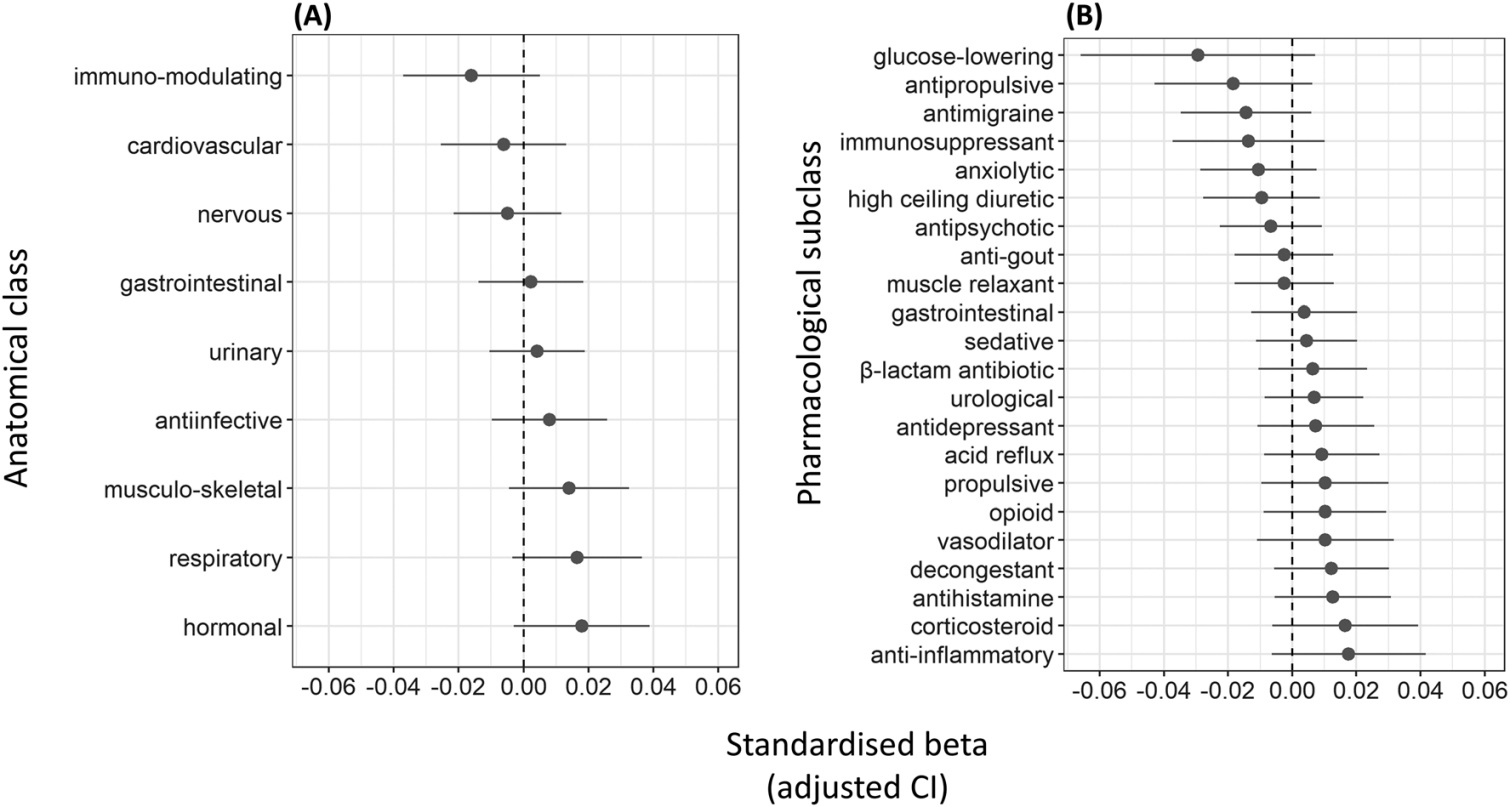
Associations between anticholinergic burden according to the scale by Durán *et al*.^52^ due to different classes of drugs on the 1 hand and total brain volume on the other. Results are displayed as standardized βs. (A) Classification of drugs based on anatomical class; (B) Classification of drugs based on pharmacological subclass. Classes with too few prescriptions in the sample (<100) were not included in the models.

AChB was also not associated with the volume of any cortical (range of β = −0.018–0.028, *P*_FDR_ ≥ .26) or subcortical (β range = −0.007–0.024, *P*_FDR_ ≥ .08) brain region, or the microstructure of white matter tracts (range of β = −0.015–0.014, all *P*_FDR_ = .98; Table S14). AChB due to no drug class was associated with brain atrophy (Table S15, Figure 4).

### 3.4 Sensitivity analyses

When the analyses on the associations between AChB and cognitive function were repeated using only AChB in the year before the assessment as the predictor (Tables S16–S19), the results exhibited similar trends to those observed in the main analyses. Most anticholinergic scales tended to negatively associate with cognitive function, albeit the effect sizes were smaller. Additionally, AChB was associated with lower performance in 1/5 cognitive tests available for this analysis. Furthermore, AChB due to β‐lactam antibiotics and opioids again exhibited the strongest negative associations with *g*.

When *g* was modelled with the inclusion of an interaction term between age at assessment and AChB, the interaction was not significant (β = 3.0 × 10^−4^, *P* = .38), indicating that the observed effect sizes were not substantially larger or smaller in older compared to younger participants.

## 4 DISCUSSION

In this study, we found that most of the 15 studied anticholinergic scales exhibited significant associations with cognitive ability. This remained the case after controlling for multiple potential confounds, including the history of certain disorders and polypharmacy. Interestingly, the size of the effect was not moderated by age—middle‐aged and older adults showed similar AChB‐cognitive associations. While the positive association between higher AChB and lower cognitive ability largely agrees with previous studies on the topic, past results have been mixed.^7^, ^16^ One potential source of heterogeneity between studies is different control for polypharmacy, which may alter the results considerably. In our study, the addition of polypharmacy substantially decreased the size of the observed effects and was a stronger predictor of lower cognitive ability than AChB according to any of the studied anticholinergic scales. Another source of heterogeneity may be the differential effect of distinct drug classes. We found large differences between drug classes when predicting cognitive ability, with β‐lactam antibiotics exhibiting larger effects than other drug classes. Moreover, antimigraine drugs were associated with higher cognitive ability. The effect of a general anticholinergic score may thus strongly depend on the structure of the sample and the precise prescribing characteristics of the participants.

In our study, general AChB was not predictive of any measure of brain structural MRI studied, including the volumes of 68 cortical and 14 subcortical areas, and measures of brain microstructure for 25 white matter tracts. These findings are in contrast with previous research. To our knowledge, 4 studies have explored the association between anticholinergic use and brain structure. They found anticholinergic use to associate with reduced cortical volume and reduced temporal lobe thickness,^12^ increased rates of brain atrophy,^8^ reduced grey matter density and functional connectivity in the nucleus basalis of Meynert,^38^ and reduced volumes of both hippocampi.^39^

It is unclear why our results from MRI structural imaging diverge from previous findings, as the studies described above display a range of characteristics that overlap with our own, including longitudinal data,^8^ control for polypharmacy,^12^ and the inclusion of middle‐aged participants.^8^, ^39^ One possibility is that the previous studies mostly classified the predictor (e.g., anticholinergic users *vs*. nonusers), while we used a continuous measure of AChB. The pitfalls of categorisation and the loss of power for true effects have been discussed before.^65^ Furthermore, the size of our imaging sample (~16 000) was several times larger (~3000). As has been recently reported,^66^ brain‐wide association studies may require thousands of participants to minimize effect size inflation and increase replication rates. Finally, all but 1^39^ of the above studies focused on cognitive disorders or decline later in life, with 1 reporting an effect for specifically those participants that later developed mild cognitive impairment.^38^ It is possible that while brain atrophy occurs in ageing or dementia, subtle cellular changes in the cholinergic system occur before that but are not measurable by structural and diffusion MRI. This could include changes in the proportions or the integrity of muscarinic receptor subtypes or a shift in the balance of oscillation frequencies of neural networks.

Our study exhibits several advantages, including the use of a far larger sample than ever before in this area, use of linked prescriptions from primary care across a long period, exploration of several outcomes, the use of a latent factor of cognitive ability, and the comparison of different anticholinergic scales and classes of drugs. Furthermore, our models carefully incorporated several important control variables, including the history of relevant disorders, polypharmacy, and several lifestyle and demographic factors. Finally, we adopted a robust approach to measuring cognitive ability that can reduce variability common in the assessment of separate cognitive domains.

However, we recognize several limitations. First, the UK Biobank sample is on average less deprived and healthier than the UK population^67^ and thus not representative. Participants in the imaging subsample exhibit even better indicators of psychological and physical health than the UK Biobank average.^68^ Both factors are likely to result in an underestimate of the effects present in the population. Second, the prescriptions included in our study do not incorporate over‐the‐counter drugs and we also have no information on how many prescriptions were dispensed or taken by participants. Third, brain imaging was sometimes performed after the coverage for prescriptions had concluded and the drugs potentially prescribed in the intervening period were not accounted for. This probably decreased the accuracy of our AChB measure for those participants. Fourth, our study was cross‐sectional and did not assess longitudinal changes in cognitive function and brain structure. This prevented us from establishing the sequence of events and from assessing associations between anticholinergic use and within‐person changes. Finally, because AchB correlates with the number of anticholinergics, the effects of polypharmacy due to the use concurrently of several anticholinergic drugs and intrinsic anticholinergic activity of those drugs could not be completely separated.

Both the present study, as well as previous analyses have reported polypharmacy more broadly to be associated with poorer cognitive ability^69^, ^70^ and dementia.^71^ A recent medication‐wide association study^72^ found that among 744 medicines, 30% were associated with dementia. Additionally, previous studies have reported on differences between drug classes in the association between AchB and dementia.^9^, ^10^, ^11^ This finding was extended in the present study of general cognitive ability in a nonpathological sample. These results support a more nuanced approach that distinguishes between different classes of drugs beyond their assumed anticholinergic effects. For drug classes for which associations with lower cognitive ability or dementia can be demonstrated, more studies are needed to determine the effects of chronic use earlier in life, the impact of discontinuation and the potential neural correlates.

In summary, in this study, we found positive associations between long‐term anticholinergic use and general cognitive ability across most studied anticholinergic scales. However, the associations held only for some drug classes and there was no evidence for differences in brain structure as a function of AChB. While the significant effect sizes observed in our study were modest, for complex, multicausal outcomes—especially in a large and relatively healthy sample—this is to be expected. For example, angiotensin converting enzyme inhibitors—one of the most common drugs to treat hypertension—have been shown to reduce systolic/diastolic pressure by merely −8/−5 mmHg.^73^ When considered in the long‐term and on the scale of entire populations, even tiny effects can accumulate to produce substantial health and economic consequences for society. Given sufficient confidence in a drug‐outcome relationship and the availability of alternative treatments, changes in prescribing represent an intervention that is relatively simple to implement. This should serve as additional motivation for further research in the field.

## COMPETING INTERESTS

R.E.M. has received consulting fees from the Epigenetic Clock Development Foundation and speaker fees from Illumina. T.C.R. has received fees for medicolegal work from private solicitors. S.R.C. has received speaker fees from the Society of Biological Psychiatry. G.M.T. has received consulting fees for grants funded by the NIH. J.M. has nothing to disclose.

## CONTRIBUTORS

J.M. conceived and planned the initial study, prepared the data, and conducted the statistical analysis. J.M. and S.R.C. contributed to the data analysis strategy. All authors contributed to the interpretation of findings, the revision of the manuscript, and the approval of the final version.

## Supporting information


**Table S1.** UK Biobank variables and their Field IDs used in the study.
**Table S2.** All cognitive tests available in UK Biobank, with the mean and standard deviation for each test (before scaling), and the numbers of participants from our sample (and % of the sample) that underwent testing at either the baseline or the imaging assessment. The greyed‐out tests were not used in our study, either due to measuring crystallised cognitive ability (Picture Vocabulary) or due to ceiling‐effects (Paired Associative Learning). The Trail Making Tests, Tower Rearranging, Matrix Pattern Completion, and Symbol Digit Substitution were not administered during the baseline assessment. The data has been cleaned for outliers, defined as values four or more standard deviations below or above the mean. *Note: Prospective memory was a binary variable; the values indicate the numbers (and %) of participants with correct recall.
**Figure S1.** Path diagram for SEM calculating the latent *g* from individual cognitive tests administered during the baseline assessment (top) or during the imaging assessment (bottom). The arrows depict standardised loadings (the latent variable and the observed variables have a variance of 1), with positive loadings depicted with grey one‐way arrows and negative loadings depicted with red one‐way arrows. The dotted line indicates that the (unstandardised) loading was fixed to 1. MR and VNR on the one hand, and DSS and RT one the other hand measure similar cognitive abilities and residual correlations between them were included in the model; this is depicted by two‐way arrows between the cognitive tests. Two‐way arrows within individual cognitive tests represent residual variances. The total variance in cognitive test scores explained by the latent factor was 0.23 for the baseline assessment and 0.28 for the imaging assessment. The model‐fit statistics are displayed on the right side of each diagram. *Note*: MR: Matrix Pattern Completion; DSS: Digit Symbol Substitution; VNR: Fluid Intelligence; TMTb: Trail Making Test B; RT: Reaction Time; VisMem: Pairs Matching; ProsMem: Prospective Memory; NM: Numeric Memory; TR: Tower Rearranging.
**Figure S2.** Cortical regions from the Desikan‐Killiany neuroanatomical atlas (top), white matter tracts (bottom left) and subcortical structures (bottom right) measured in the present study. Figures reused from previous studies.^1,2^ *Note*: AR, acoustic radiation; ATR, anterior thalamic radiation; Cing, cingulum (gyrus and parahippocampal); CST, corticospinal tract; Fmaj and Fmin (forceps major and minor); IFOF, inferior fronto‐occipital fasciculus; ILF, inferior longitudinal fasciculus; MCP, middle cerebellar peduncle; ML, medial lemniscus; PTR, posterior thalamic radiation; SLF, superior longitudinal fasciculus; STR, superior thalamic radiation; Unc, uncinate fasciculus.
**Table S4.** Anticholinergics scales identified in the present study. We considered anticholinergic scales that were available as complete lists of drugs, scored each drug for its anticholinergic potency, and did not require information on dosage. Grey shading indicates that the scale was not considered for further analysis. For two scales,^3,4^ updated versions (Aging Brain Care, 2012; Carnahan, 2014, personal communication on 21.10.2019) were used. One scale^5^ was updated to include newer drugs from the UK market as has been done before.^6^ The table was modified from a previous study.^7^
**Figure S3.** The data‐cleaning pipeline. The upper and lower rows of numbers represent the numbers of observation and participants, respectively. The rectangular boxes display the numbers of observations/participants at each point of the data‐cleaning process, while the ovals display the numbers of observations/participants removed. The colour of the rectangles indicates the type of observation: in the grey boxes, the basic observation was a single prescription; in the blue boxes, it was a year‐participant pair, with each pair representing the AChB of a single participant in a given year. The actions performed at each step are written above the arrows and signify the (1) removal of prescriptions without any content (i.e., no drug indicated), (2) removal of prescriptions without dates or impossible dates (e.g., in the future or far in the past), (3) separation of combination drugs into individual compounds, (4) removal of prescriptions that appear after the death of the participant, (5) transformation into the year‐participant format, (6) removal of observations (generated in step (5)) occurring after the end of the prescription‐sampling period for any participant, (7) removal of observations for the first year in the dataset for each participant (as it is unlikely to be complete), (8) removal of observations prior to the year 2000 and after the year 2015, (9) removal of observations for participants diagnosed with a disorder that may affect cognitive or brain function, (10) removal of observations after or within the year of the UK Biobank assessment.
**Figure S4.** Q‐Q plots of theoretical quantiles (x‐axis) *vs.* standardised residuals (y‐axis) for some models used in our study. Only the examples of models exhibiting most extreme kurtosis are shown. The examples here are for the association between AChB according to Durán *et al*. (2013)^19^ on the one hand and either *g* (1), reaction time (2), visual memory (3), volume of the right pastriangularis (4), volume of the left pallidum (5), or volume of the right hippocampus (6), on the other.
**Figure S5.** Scree plots of eigenvalues for FA (left) and MD (right). The principal component for FA explained 43.9% of the total variance and the principal component for MD explained 49.9% of the total variance.
**TEXT S1.** The models used in our study. (1.0) was used to compare anticholinergic scales (with each model using *g* as the outcome) and to compare cognitive tests (with each model using the scale by Durán *et al*. (2013) ^19^ as the predictor and a different cognitive test as the outcome). (1.1) is the basic polypharmacy model; it differs from (1.0) in that the number of non‐anticholinergic drugs is the main predictor. (1.2) is the Polypharmacy Plus model that differs from (1.1) in that the number of anticholinergic drugs (according to any anticholinergic scale) is included as a covariate. (2.0) comprises of models that predict any measure of brain‐MRI by AChB (according to the scale by Durán *et al*. (2013) ^19^ when not comparing scales) and include additional covariates. Polypharmacy models (analogous to 1.1 and 1.2, but with additional covariates as in 2.0) were also run for total brain volume when comparing anticholinergic scales.
**Table S6.** Demographic and lifestyle characteristics of separately the imaging subsample and the rest of the sample after the removal of outliers. The columns indicate the median and IQR (or n and % for categorical variables) and the number of missing observations. The variables are not scaled. Note that the counts may not always add up to those depicted in Table 1, as the imaging subsample underwent separate data‐cleaning before running the analyses.
**Table S7.** Numbers (and % of total prescriptions) of anticholinergic prescriptions per anticholinergic scale and numbers (and % of total sample) of participants prescribed at least one anticholinergic prescription in the sampling period. The data include the period between the year 2000 and the year prior to attending the assessment visit (differs between participants). *Note: some scales were updated after their initial date of publication, as noted in Supplementary Table 3.
**Figure S6.** Scatterplot for the association between the number of drugs identified as having an anticholinergic effect and the association with lower cognitive ability. The x‐axis represents the number of drugs identified as possessing anticholinergic effects, the y‐axis represents the absolute value of the standardised β for the association between AChB and *g*. Each dot in the scatterplot represents an anticholinergic scale. The red line represents the line of best fit, with grey shading indicating the 95% CI.

**Table S3.** Standardised loadings for each cognitive test for the g calculated from tests from the baseline assessment (above) and for the g calculated from tests from the imaging assessment (below). *Note: MR: Matrix Pattern Completion; DSS: Digit Symbol Substitution; VNR: Fluid Intelligence; TMTb: Trail Making Test B; RT: Reaction Time; VisMem: Pairs Matching; ProsMem: Prospective Memory; NM: Numeric Memory; TR: Tower Rearranging.

**Table S5.** Standardised loadings for the first principal component (PC1) of FA and MD.

**Table S8.** Results for linear models on the association between AChB and *g*. The columns show the standardised betas, FDR‐adjusted p‐values, and numbers of missing/excluded observations (from the total of 163,043) for the different anticholinergic scales. For each anticholinergic scale, test statistics are shown for models excluding and including non‐anticholinergic polypharmacy as a control variable. The latter is computed by subtracting the number of anticholinergic prescriptions from the total number of prescriptions. For the two models of polypharmacy (bottom of the table), only the main predictor (non‐anticholinergic drug count, i.e., polypharmacy) is displayed.

**Table S9.** Results for linear models on the association between AChB according to the scale by Durán *et al*. (2013)^19^ and *g*. The columns show the standardised betas and p‐values. *Note: BMI: body mass index; TPP: The Phoenix Partnership.

**Table S10.** Results for linear models on the association between AChB according to the scale by Durán *et al*. (2013)^19^ and individual tests of cognitive function. The columns show the abbreviated names of the cognitive tests, standardised betas, FDR‐adjusted p‐values, and the numbers of missing/excluded observations (from a total of 163,043). *Note: DSS: digit Symbol Substitution test; TMTb: Trail‐Making test; TR: Tower Rearranging test; ProsMem: Prospective Memory; VNR: Fluid Intelligence; MR: Matrix Pattern Completion; NM: Numeric Short‐Term Memory; VisMem: Pairs‐Matching test; RT: Reaction Time.

**Table S11.** Results for linear models on the association between AChB according to the scale by Durán *et al*. (2013)^19^ and *g*. The columns show the classification system according to the WHO‐ATC (1: anatomical main group; 3: pharmacological subgroup), standardised betas, thresholds for significance after FDR correction, FDR‐adjusted p‐values, and numbers of missing/excluded observations (from a total of 163,043) for the different drug classes. Classes of drugs prescribed to too few individuals in the sample (<100) were not included in the models.

**Table S12.** Results for linear models on the association between AChB and total brain volume. The columns show the standardised betas, FDR‐adjusted p‐values, and numbers of missing/excluded observations (from the total of 17,337) for the different anticholinergic scales.

**Table S13.** Results for linear models on the association between AChB according to the scale by Durán *et al*. (2013)^19^ and total brain volume. The columns show the standardised betas and p‐values. *Note: BMI: body mass index; TPP: The Phoenix Partnership.

**Table S14.** Results for linear models on the association between AChB according to the scale by Durán *et al*. (2013)^19^ and various measures of structural MRI: volume of cortical areas (A), volume of subcortical areas (B), fractional anisotropy (C), and mean diffusivity. The columns show the standardised betas, FDR‐adjusted p‐values, and numbers of missing/excluded observations (from the total of 17,337) for the different measures.

**Table S15.** Results for linear models on the association between AChB according to the scale by Durán *et al*. (2013)^19^ and total brain volume. The columns show the classification system according to the WHO‐ATC (1: anatomical main group; 3: pharmacological subgroup), standardised betas, FDR‐adjusted p‐values, and numbers of missing/excluded observations (from a total of 17,337) for the different drug classes. Classes of drugs prescribed to too few individuals in the sample (<100) were not included in the models.

**Table S16.** Results for linear models on the association between AChB in the year before the assessment and *g*. The columns show the standardised betas, FRD‐adjusted p‐thresholds, FDR‐adjusted p‐values, and numbers of missing/excluded observations (from the total of 163,043) for the different anticholinergic scales. For each anticholinergic scale, test statistics are shown for models excluding and excluding polypharmacy as a control variable. The latter is computed by subtracting the number of anticholinergic prescriptions from the total number of prescriptions. For the two models of polypharmacy (bottom of the table), only the main predictor (non‐anticholinergic drug count, i.e., polypharmacy) is displayed. The columns show the author (and year of publication) of the scale, whether polypharmacy was included in the model as a covariate, standardised beta, FDR‐corrected p‐values, and the number of missing/excluded observations.

**Table S17.** Results for linear models on the association between AChB according to the scale by Durán *et al*. (2013)^19^ calculated in the year before the assessment and *g*. The columns show the standardised betas and p‐values. *Note: BMI: body mass index; TPP: The Phoenix Partnership.

**Table S18.** Results for linear models on the association between AChB according to the scale by Durán *et al*. (2013)^19^ calculated for the year before the assessment and individual tests of cognitive function. The columns show the abbreviated names of the cognitive test, standardised betas, FDR‐adjusted p‐values, and the numbers of missing/excluded observations (from a total of 147,719). DSS: digit Symbol Substitution test; TMTb: Trail‐Making test; TR: Tower Rearranging test; ProsMem: Prospective Memory; VNR: Fluid Intelligence; MR: Matrix Pattern Completion; NM: Numeric Short‐Term Memory; VisMem: Pairs‐Matching test; RT: Reaction Time.

**Table S19.** Results for linear models on the association between AChB according to the scale by Durán *et al*. (2013)^19^ calculated for the year before the assessment and *g*. The columns show the classification system according to the WHO‐ATC (1: anatomical main group; 3: pharmacological subgroup), standardised betas, FDR‐adjusted p‐values, and numbers of missing/excluded observations (from a total of 147,719) for the different drug classes. Classes of drugs prescribed to too few individuals in the sample (<100) were not included in the models.

## Data Availability

Data from UK Biobank is available to approved researchers directly from UK Biobank. The code used to clean and analyse the data is available at https://github.com/JuM24/UKB-AChB-cognition-MRI.
